# Role of PSMA PET-guided metastases-directed therapy in oligometastatic recurrent prostate cancer

**DOI:** 10.3389/fonc.2022.929444

**Published:** 2022-08-18

**Authors:** Matthew Alberto, Arthur Yim, Nathan Papa, Shankar Siva, Joseph Ischia, Karim Touijer, James A. Eastham, Damien Bolton, Marlon Perera

**Affiliations:** ^1^ Department of Surgery, Austin Health, University of Melbourne, Melbourne, VIC, Australia; ^2^ Department of Preventive Medicine, Monash University, Melbourne, VIC, Australia; ^3^ Department of Radiation Oncology, Peter MacCallum Cancer Centre, Melbourne, VIC, Australia; ^4^ Urology Service, Department of Surgery, Memorial Sloan Kettering Cancer Center, New York, NY, United States

**Keywords:** prostate cancer, PSMA PET, metastatic disease, oligometastatic, metastastases directed therapy

## Abstract

Oligometastatic prostate cancer (OMPC) has been proposed as an intermediary state between localised disease and widespread metastases, with varying definitions including 1, 3, or ≤5 visceral or bone metastasis. Traditional definitions of OMPC are based on staging with conventional imaging, such as computerised tomography (CT) and whole-body bone scan (WBBS). Novel imaging modalities such as prostate-specific membrane antigen positron emission tomography (PSMA PET) have improved diagnostic utility in detecting early metastatic prostate cancer (PC) metastases compared with conventional imaging. Specifically, meta-analytical data suggest that PSMA PET is sensitive in detecting oligometastatic disease in patients with biochemical recurrence (BCR) post-radical treatment of PC. Recent trials have evaluated PSMA PET-guided metastases-directed therapy (MDT) in oligometastatic recurrent disease, typically with salvage surgery or radiotherapy (RT). To date, these preliminary studies demonstrate promising results, potentially delaying the need for systemic therapy. We aim to report a comprehensive, multidisciplinary review of PSMA-guided MDT in OMPC. In this review, we highlight the utility of PMSA PET in biochemically recurrent disease and impact of PSMA PET on the definition of oligometastatic disease and outline data pertaining to PSMA-guided MDT.

## Introduction

Prostate cancer (PC) is the second most frequent cancer (37.5 per 100,000) and the fifth most common cause of cancer death in men (1). With significant burden of disease, PC management has steadily advanced throughout the years with improved treatment pathways for primary localised disease, locally advanced disease, oligometastatic cancer, and high-volume metastatic disease. Despite a shift towards early diagnosis with the introduction of screening with prostate-specific antigen (PSA) (2), a proportion of cases harbour an aggressive disease course. Optimal treatment pathways for patients with localised high-risk disease are ill-defined, but broadly, these patients may receive prostatectomy or radiotherapy (RT)—which may provide oncological control and improvements of local symptoms (3). However, the risk of recurrent disease exists in these patients, even when treated during the localised disease course. Indeed, previous high-volume series suggests a 50% risk of biochemical recurrence and 37% risk of salvage therapy 10 years after prostatectomy in high-risk patients (4)

In the setting of disease recurrence, oligometastatic PC (OMPC) can be considered an intermediary state between localised disease and widespread metastases with heterogeneous definitions including 1, 3, or ≤5 visceral or bone metastasis (5–10). Given the limited deposits of metastatic disease, several groups proposed consideration of metastasis-directed therapy (MDT) with the aim of optimising oncological outcomes (11). However, most MDT has been based on conventional imaging with computed tomography (CT), ^99m^Tc-methylene diphosphonate (MDP) bone scintigraphy, and choline-positron emission tomography (PET) (12–15).

More recently, novel imaging techniques have been developed including prostate-specific membrane antigen (PSMA) PET (16). Of these, the most widely studied radioligands include ^68^Ga-PSMA-11 (17) and ^18^F-PSMA-DCFPyL (18). These techniques have provided improved diagnostic accuracy in the management of advanced PC, especially in biochemical recurrence (BCR) (17, 19, 20). Specifically, PSMA PET techniques allow visualisation of metastatic disease prior to metastatic deposits, reaching morphological criteria required for diagnosis on conventional imaging (21). Hence, recent trials have evaluated PSMA PET-guided MDT in oligometastatic recurrent disease, typically with salvage surgery or radiotherapy, which potentially delays the need for systemic therapy (22–24). Therefore, we aim to comprehensively review PSMA PET-guided MDT in OMPC. Our report will highlight PSMA PET in BCR and paradigm shifting definitions of oligometastatic disease and discuss current trials in PSMA PET-guided MDT.

## The role of PSMA PET in detection of biochemically recurrent disease

PSMA is a cellular surface protein with high expression in prostate tissue and limited extraprostatic expression. It is a 750-amino-acid, 100-kDa, type II transmembrane glycoprotein consisting of intracellular, transmembrane, and extracellular components (25). It may also be expressed in other organs including the kidney, small bowel, neuroendocrine tissue, and neural tissue (26). However, PSMA has been found to have 12 times greater expression on prostatic tissue compared to the next highest organ (27). Furthermore, studies have demonstrated PSMA expression in dysplastic prostatic changes and subsequent marked expression in prostatic adenocarcinoma and lymph node metastases (LNMs), whilst it is lowest in benign prostatic tissue (28, 29). Increased PSMA expression also occurs in the setting of increasing grade and stage of PC (26, 30). Additionally, in oligometastatic disease, only 2% of LNMs have been found to be negative for PSMA expression (29). Hence, PSMA represents an attractive target for imaging and therapeutic intervention in PC.

Following radical therapy, such as prostatectomy or radiotherapy, PSMA PET in the setting of BCR has been extensively investigated; however, definitions of BCR have been varied. A prostate-specific antigen (PSA) > 0.4 ng/ml and rising has been noted to best predict further metastases after radical prostatectomy (RP) (31, 32), although a PSA ≥ 0.2 ng/ml and confirmed on subsequent check post-RP has also been proposed (33). Hence, the European Association of Urology (EAU) Prostate Cancer Guidelines Panel recommends evaluating a patient’s life expectancy when considering further treatment and should not be based on meeting a PSA threshold. Rather, the EAU suggests utilising an externally validated, patient-specific risk stratification system dividing into EAU Low-Risk BCR [PSA-doubling time > 1 year and Primary Gleason Score <8 (ISUP grade < 4) for RP] and EAU High-Risk BCR [PSA-doubling time ≤ 1 year or Primary Gleason Score 8-10 (ISUP grade 4–5)] (34). Furthermore, the EAU guidelines recommend early restaging and early immediate post-operative RT in high-risk BCR.

With the definition of biochemical recurrence in mind, a systematic review and meta-analysis of 37 articles involving 4,790 patients by Perera et al. (17, 19) noted 76% overall percentage positivity for ^68^Ga-PSMA PET in BCR. Increasing risk of positivity was associated with increasing post-treatment PSA. Specifically, for PSA between 0 and 0.19, 0.2 and 0.49, 0.5 and 0.99, and 1 and 1.99 or ≥2 ng/ml, the proportion of positive PSMA PET was 33%, 45%, 75% and 95%, respectively. These articles demonstrate the usefulness of PSMA PET in the setting of BCR PC, particularly at low levels of pre-PET PSA >0.2 ng/ml.

However, head-to-head comparison of novel imaging with conventional staging (CT and ^99m^Tc-MDP bone scintigraphy) is limited, particularly in the biochemically recurrent setting. In the primary staging setting, Hofman et al. highlighted superiority of PSMA PET versus conventional imaging in a randomised open-label cross-over trial (21). In the setting of BCR, a recent prospective single-centre clinical trial by Joshi et al. (35) compared PSMA PET and MRI with conventional imaging in 30 patients with BCR following radical curative therapy for PC. Histological correlation was performed to assess clinical efficacy of PSMA PET. Median PSA was 0.69 ng/ml, and PSMA avid lesions were present in 21 patients (70%) compared to 5 patients in conventional imaging (17%) (35). Detection of local recurrence was significantly more likely in PSMA PET/MRI when compared to conventional imaging (*p*=0.005) and eight of nine biopsied lesions were positive (88.9%) for metastatic PC with a positive predictive value of 95.2% (35). Eissa et al. (20) further corroborated these findings, noting superiority of PSMA PET to conventional imaging techniques including CT and magnetic resonance imaging (MRI). Furthermore, Fendler et al. (36) retrospectively investigated 200 patients with non-metastatic castrate-resistant PC for which 55% had M1 disease on PSMA PET despite negative conventional staging, emphasising superiority of PSMA PET.

## Defining oligometastatic prostate cancer and impact of PSMA imaging

Oligometastatic cancer was first hypothesised by Hellman and Weichselbaum (37) determining the oligometastatic state as a subgroup of patients with potentially curable and limited number of metastases, hence defined as intermediary between localised and widespread metastases. Traditional definitions of OMPC are based on conventional imaging, such as CT and ^99m^Tc-MDP bone scintigraphy. OMPC can be biologically divided into *de novo* (metastatic synchronous) tumour at the time of diagnosis as compared to oligorecurrent disease (post-treatment of the primary cancer) and oligoprogressive disease and development of a second primary tumour (metachronous) (38). Aggressive management of OMPC as a distinct disease state is at the forefront of improving patient survival of an otherwise poorly prognosticated disease, hence the need for a clear definition. However, a universal definition for oligometastatic (recurrent) PC is lacking, with maximum number and location being deliberated.

Varying definitions including ≤3 or ≤5 visceral or bone metastasis have been proposed (**Table 1**) (5). Tabata et al. (6) and Ahmed et al. (7) utilised ≤5 to define OMPC; however, it differed on the location (bone only vs. not specified) and imaging modality (^99m^Tc-MDP bone scintigraphy vs. ^11^C-choline PET, MRI, CT, or combined), respectively. Ost et al. (8), Decaestecker et al. (9), and Berkovic et al. (10) defined OMPC as ≤3, but differed based on location (any vs. bone or LNs vs. bone or LNs) and imaging modality (^18^F-fluorodeoxyglucose [FDG] PET-CT or ^18^F-choline PET-CT vs. ^18^F-FDG PET-CT or ^18^F-choline PET-CT vs. ^99m^Tc-MDP bone scintigraphy and ^18^F-FDG PET-CT or ^99m^Tc-MDP bone scintigraphy and ^11^C-choline-CT), respectively.

**Table 1:** Representative historical definition of oligometastatic disease in OMPC.

Study	Type	Sample size (n)	Definition	Location	Imaging modality
Tabata et al. (6)	Retrospective	35	≤5	Bone	^99m^Tc-MDP bone scintigraphy
Ahmed et al. (7)	Prospective	21	≤5	Any	^11^C-choline PET-CT, MRI, CT or combined
Ost et al. (8)	Prospective	119	≤3	Any	^18^F-FDG PET-CT or ^18^F-choline PET-CT
Decaestecker et al. (9)	Prospective	50	≤3	Bone or LNs	^18^F-FDG PET-CT or ^18^F-choline PET-CT
Berkovic (10)	Prospective	24	≤3	Bone or LNs	^99m^Tc-MDP bone scintigraphy and ^18^F-FDG PET-CT or ^99m^Tc-MDP bone scintigraphy and ^11^C-choline-CT

LN, lymph node; MDP, methylene diphosphonate; MRI, magnetic resonance imaging; FDG, fluorodeoxyglucose; PET, positron emission tomography; CT, computed tomography.

Due to the heterogeneity of OMPC definitions, more recent clinical trials take into account the disease burden, stratifying into low and high risk/volume as defined in the LATITUDE (39) and CHAARTED (40) criteria. The LATITUDE trial defined high risk as having two or more of the following criteria: ≥3 bone metastases, visceral metastases, and ≥ ISUP grade 4. The CHAARTED trial defined high-volume disease as ≥4 bone metastases (including ≥1 in vertebral column or spine) or visceral metastases. Therefore, OMPC can be defined as low risk and low volume based on the noted criteria in hormone-sensitive PC, which aids in the determination of treatment, such as RT to the primary for low-volume disease (41). However, these two trials also utilised conventional imaging in identifying metastatic disease. Novel imaging, such as PSMA PET, has subsequently changed the definition of OMPC due to its significant detection of disease at low pre-PET PSA. Interestingly, Barbato et al. (42) attempted to combine the CHAARTED low-/high-volume disease criteria on PSMA PET compared to CT, using 40 ml as an arbitrary cutoff. PSMA PET was concluded to have improved tumour volume assessment due to detection of additional lesions in 62% of patients. However, the study was retrospective and had low numbers (n=105) and omitted bone scintigraphy, highlighting the need for larger studies.

As noted, definitions of OMPC can be classified based on biology (*de novo* vs. oligorecurrent vs. oligoprogressive), location (visceral vs. bone vs. both), and volume/risk (low vs. high). Diagnosis of nodal metastatic disease, historically, is based on achieving morphological criteria such as size criteria >10 mm based on conventional imaging. PSMA PET has enabled earlier detection of oligometastatic disease when compared to conventional imaging and may detect lymph node metastases <10 mm. As such, PSMA PET detection rates of OMPC compared to conventional imaging was significantly higher (*p*=0.005) with a positive predictive value of 95.2% (35) and best at pre-PET PSA levels of >0.2 ng/ml (17). Furthermore, the earlier diagnosis of OMPC must be considered with caution. For example, patients with subcentimetre PSMA avid nodes diagnosed as OMPC may have previously deemed localised disease on conventional imaging. Accordingly, it is probable that the OMPC population diagnosed on PSMA PET is impacted by the Will Rogers’ stage-migration phenomenon. Moreover, with earlier detection of metastatic disease with PSMA PET, we may be artificially observing prolonged overall survival through lead-time bias (43). Despite earlier detection, the natural history of disease course may not be altered. Although more and more studies are investigating the use of metastasis-directed therapy (MDT), correction of lead-time bias is important to gain accurate measurement of improvement in overall survival.

## Historic data on MDT in the pre-PSMA era

Given that OMPC is considered an intermediate state of tumour spread with limited metastatic capacity (44), the importance of oligometastatic disease is increasingly acknowledged, as evidence grows for the treatment of limited metastatic lesions. The rationale for MDT in oligometastatic cancer can be addressed twofold—biologically and clinically. From a biological standpoint, overall reduction in tumour burden (cytoreductive therapy) may explain improved outcomes with treatment of primary and metastatic sites (45), whereas clinically, MDTs may potentially delay further metastatic progression and postpone the use of systemic treatment, reducing the burden of adverse drug effects, as this is indeed true for other tumour types such as colorectal cancer, sarcomas, and renal cell carcinoma (13). Focal ablative therapies, such as stereotactic body radiation therapy (SBRT), surgery, or focal thermal ablation, are examples of MDTs (11).

The success of MDTs relies on imaging modalities with high diagnostic accuracy to sensitively guide targeted therapy. However, conventional imaging with CT and ^99m^Tc-MDP bone scintigraphy demonstrates poor sensitivity to detect oligometastatic disease (12). The advent of modern imaging techniques including whole-body MRI and PET/CT scans using tracers, such as ^18^F-NaF or ^18^F-choline, have been frequently incorporated into guidelines and trials in recent years (12). Indeed, prior to the widespread use of PSMA PET/CT, MDTs for OMPC were predominantly diagnosed with choline PET/CT, and the literature consists of small heterogeneous studies. In a systematic review by Ost et al. (13), a total of 450 patients were pooled from 15 single-arm case series, whereby PET/CT was used for diagnosis in 98%, using either choline (91%) or FDG (7%) as tracer. Treated metastases were predominantly nodal (78%), bone metastases (21%), and less frequently visceral metastases (1%). MDT modality was either high-dose radiotherapy (66%) or surgical metastasectomy (34%). Although there was great heterogeneity among patient populations, the authors found that 51% of men were progression free 1–3 years after MDT. Results should be interpreted with caution, however, as 61% had adjuvant androgen deprivation therapy (ADT) and 49% had adjuvant nodal irradiation. Due to the overall low number and heterogeneity of patients, and lack of comparative or randomised trials, the review concluded that MDT should not be considered the standard of care. In an attempt to overcome the limitations of retrospective studies, a multi-institutional analysis used fixed inclusion and exclusion criteria, demonstrating an ADT-free survival of 28 months after SBRT for oligorecurrent PC (8). However, 50% of these patients also received a temporary course of adjuvant ADT at the time of SBRT.

There are several small case series that demonstrate benefit of MDT without ADT, showing a median progression-free survival of 24 and 19 months following SBRT (9, 46), and 4 years following salvage LND (47). Recently, the Surveillance or Metastasis-Directed Therapy for Oligometastatic Prostate Cancer Recurrence (STOMP) trial aimed to validate the observations of previous retrospective studies showing benefits of MDT (14). STOMP was the first prospective, randomised, phase II trial that demonstrated that MDT using SBRT delayed the initiation of ADT in men with hormone-sensitive metastatic PC with ≤3 detectable metastases on choline PET/CT. The primary outcome of the STOMP trial was ADT-free survival in men assigned to MDT versus surveillance alone. The results showed that those undergoing MDT experienced a longer median ADT-free survival of 21 months compared to 13 months in the surveillance arm [HR, 0.60 (0.40–0.90, 80% CI), log-rank *p*=0.11]. At the last update, the 5 year ADT-free survival was 34% for the MDT group and 8% for the surveillance group (15). However, 30% of patients treated with MDT progressed to polymetastatic disease (>3 metastases) within the first year. Authors suggest that this may be due to microscopic metastatic disease, which is not seen by choline PET/CT, but may be overcome in future studies utilising PSMA PET/CT, which has much greater sensitivity and specificity (14).

Stereotactic ablative body radiotherapy (SABR) was utilised by Siva et al. (48), who performed the second prospective trial demonstrating delayed initiation of ADT on 33 patients with one to three metastases utilising NaF PET/CT. A single fraction of 20-Gy SABR was prescribed to 50 lesions and reviewed with imaging at 12 and 24 months. Local progression-free survival was 97% (91%–100%, 95% CI) at 12 months and 93% (84%–100%, 95% CI) at 24 months, and distant progression-free survival was 58% (43%–77%, 95% CI) at 12 months and 39% (25–60%, 95% CI) at 24 months. As opposed to the STOMP trial, ADT was initiated on clinician discretion instead of pre-defined protocols and was delayed by 24 months in 48% (31%–75%, 95% CI). The authors also note the low sensitivity and specificity of NaF PET for the detection of nodal metastases, which may exclude truly oligometastatic disease patients.

Another treatment modality for patients with recurrent oligometastatic disease is salvage lymph node dissection (SLND). A retrospective study by Rischke et al. (49) found that adjuvant RT delayed BCR in 93 patients who underwent SLND in comparison to SLND alone with the 5-year relapse-free rate of 70.7% vs. 26.3% (p<0.0001), respectively. However, given the small numbers, retrospective analysis, and superseded imaging modality for the identification of oligometastases (^11^C or ^18^F PET/CT), the authors suggest that prospective randomised trials are required for confirmation of adjuvant RT.

## Current data on MDT guided by PSMA

Given the sensitivity of PSMA PET for the localization of sites of recurrence, this new emerging imaging modality has the potential to directly impact MDT in several ways. Not only will it redefine the treatment paradigms for oligometastatic disease, but it will also allow MDT to target involved areas that would not normally be included on historical consensus guidelines (50). In the post-operative setting, a recent retrospective multicentre study evidenced the high detection rate (from 40.9% for a PSA value range of 0.2–0.4 ng/ml to 64.2% for a value in the range from 0.8 to 1 ng/ml) of ^68^Ga-PSMA PET/CT in a population of early biochemical recurrence, with PSA ≤1 ng/ml after radical prostatectomy. These results suggest that PSMA imaging pre-salvage radiotherapy might significantly influence disease management of early biochemical recurrence, with PSMA PET guiding optimal clinical approach (51). Additionally, in a recent multicentre analysis of 270 patients who presented for salvage radiotherapy, 19% of patients had at least one lesion identified on PSMA PET/CT that was outside a consensus prostate fossa + pelvic lymph node radiation field, with 12% overall having extra-pelvic disease (52).

In a recent systematic review of next-generation imaging modalities of recurrent oligometastatic disease, PSMA PET-directed salvage therapy was used in 50% of studies (22). Of note, in the recent phase II Observation vs. Stereotactic Ablative Radiation for Oligometastatic Prostate Cancer (ORIOLE) trial, randomised 80 patients received SBRT vs. observation alone (23). SBRT planning was based on conventional imaging alone; although a ^18^F-DCFPyL-PSMA PET was performed, it was not used for treatment planning. PSMA PET-positive lesions that were not prescribed in the treatment fields were found in 44% of patients. A *post-hoc* analysis based on the extent of untreated disease seen on PSMA PET found progression-free survival advantages for men who had received treatment to all PSMA-avid disease (HR, 0.26; 95% CI, 0.09–0.76; *p*=0.006) (23). In another recent retrospective multicentre study comparing choline-PET with PSMA PET-directed MDT, disease-free survival rates were 34% (n=15) and 64% (n=28) (*p*=0.06), respectively (24). The ADT administration rate was also higher after choline PET-guided SBRT due to the higher incidence of polymetastatic disease after first-course SBRT compared with ^68^Ga-PSMA-based SBRT (20 vs. 5 patients, *p*=0.001). Furthermore, a large multicentre retrospective study evaluated 394 patients with oligorecurrent disease comparing ^68^Ga-PSMA PET/CT-directed RT to combined elective RT (RT to prostate bed and pelvic and para-aortic nodes) plus focal therapy (53). Biochemical recurrence-free survival was significantly more in the combined PSMA PET directed and elective RT group compared with PSMA PET-directed therapy alone at 36 months (53% vs. 37%, *p*=0.001). These studies suggest that PSMA PET may delay the initiation of systemic ADT and prolong progression-free survival.

SLND represents another treatment option for patients with recurrent oligometastatic disease. However, only retrospective studies are available for the evaluation of PSMA-directed SLND. A recent systematic review highlighted that most are single-centre series with small and highly heterogeneous cohorts in terms of endpoints, adjuvant treatments, and definitions of progression (54). Of 27 studies included in the review, the majority (15/27) used choline as a tracer, whilst PSMA-labelled radionuclides were used in 11/27 (54). In the first evaluation of PLND in OMPC detected by ^68^Ga-PSMA PET, diagnostic accuracies per nodal lesion showed a sensitivity and specificity of 94% and 99%, respectively, in a total of 213 nodes from 35 patients (55). A retrospective series by Linxweiler et al. (56) compared SLND directed by ^68^Ga-labelled PSMA versus choline PET/CT, demonstrating an improvement in biochemical complete response rate (44% vs. 18%), a greater PSA decrease (mean −57% vs. mean +10%, *p *= 0.015), and a longer ADT-free period (4.7 vs. 12 months, *p*=0.001). Further advancements in minimally invasive approaches such as laparoscopic robotic-assisted SLND may also improve perioperative morbidity compared with a standard open surgical approach; however, only small retrospective case series have been published where ^68^Ga-PSMA PET (56–59) and ^99m^Technetium-PSMA PET were used (60). Interestingly, Bravi et al. (61) found on long-term follow-up (10 years) of patients with lymph node recurrence on ^11^C-chole or ^68^Ga-labelled PSMA PET/CT that one in three men treated with SLND died due to PC in the setting of PET-detected nodal PC recurrence. The authors concluded that a multimodal approach including use of ADT to maximise patient outcomes and MDT may be curative in a select population of patients.

A recent prospective phase II study by Glicksman et al. (62) used ^18^F-DCFPyL-PSMA PET to identify patients with oligorecurrent PC in the setting of rising PSA (0.4–3.0 ng/ml) post-definitive therapy (RP and post-operative RT). Out of 72 patients, 38 (53%) were found to have PSMA-detected oligorecurrent disease amenable to MDT. Ten patients underwent surgery, 27 had SABR, and 1 patient was not based on discussions with a urologist and radiation oncologist. For those treated with MDT, 60% (n=22) of patients had a biochemical response (7 surgery and 15 SABR) with 22% (n=8) meeting undetectable PSA levels (complete biochemical response) and a median follow-up duration of 7.7 months. Although this study presents promising results, data cannot be extrapolated to patients with BCR post-RP alone.

A novel surgical approach to patients with recurrent disease on ^68^Ga-PSMA PET/CT post-RP, Li et al. (63) enrolled 19 patients into integrated indocyanine green (ICG)-guided fluorescent laparoscopic SLND. The authors aimed to use ICG-guided SLND to effectively remove affected LNs and to minimise complications. The specificity of ^68^Ga-PSMA PET/CT was 96.6% (and a sensitivity of 42.9%), whilst ICG had a sensitivity of 92.8% (and a specificity of 39.1%). The authors concluded that in patients with BCR with recurrent lymph node disease, a combined approach with ^68^Ga-PSMA PET/CT and ICG fluorescence-guided SLND is an effective and safe treatment; however, further validation and long-term results are warranted.

In contrast to the high level of evidence supporting PSMA-targeted PET for post-RP BCR, fewer studies have investigated the post-primary RT population. Meta-analyses have pooled post-RT and post-RP patients together, with majority being post-RP (17, 64). In a prospective study comparing ^18^F-DCFPyL PSMA-targeted PET restaging to conventional imaging exclusively in post-RT patients, PSMA PET was able to detect more recurrence at any site [87% (78%–94%, 95% CI) vs. 67% (56%–77%, 95% CI)] and extra-prostatic sites [39% (28%–51%, 95% CI) vs. 19% (11%–29%, 95% CI), *p*<0.001] (65). The distribution of disease detected on PSMA PET was 48% prostatic, 27% regional nodes, and 30% distant. Interestingly, this differs from post-RP BCR, where failures tend to be regional nodal or extra-pelvic, with a smaller proportion exhibiting isolated prostate bed recurrence (66). The high rates of extra-prostatic disease detected in patients who meet Phoenix criteria (PSA rise ≥2 ng/ml above nadir) (67) suggest that there may be a role for PSMA-targeted PET at earlier time points post-RT to maximize detection of local failure.

## Future directions and active clinical trials

As demonstrated in an international meta-analysis on OMPC recurrence (≤3 lesions), the majority of patients treated with SBRT for nodal recurrence had a relapse within 2 years in nearby lymph node regions, with an estimated median time of 12–18 months (8). Similar results were seen in a large multi-institutional study exploring the role of SLND after nodal recurrence (59). PEACE V-Salvage Treatment of OligoRecurrent nodal prostate cancer Metastases (STORM) is a randomised phase II study aiming to assess the potential of combined whole pelvic radiotherapy and MDT against MDT alone (68). Another single-centre study (NCT04271579) (ProsTone) aims to investigate whether a unilateral pelvic lymph node dissection on the side of conspicuous PSMA PET is sufficient, without the need to perform a dissection of the contralateral side. SLND may also be carried out with the aid of experimental preoperative labelling with PSMA ligands for easier intraoperative localisation (PSMA radio-guided surgery); a comparison of conventional salvage surgery and the PSMA-radioguided surgery is also planned (NCT04271579). There are currently no randomised studies on oncological outcomes for patients who received MDT for BCR based on PSMA-targeted PET compared to other imaging. However, randomized phase III trials are ongoing (NCT03582774 and NCT03762759), with estimated completion dates in 2023 and 2025. Another emerging trend is the use of ADT and/or pelvic nodal RT combined with salvage prostate bed RT. For example in SPPORT, freedom from progression was superior in men who received RT plus 6 months of ADT (69).

In addition to diagnostic uses, PSMA-targeting agents are also being used therapeutically in a field called theranostics by utilising radiopharmaceuticals (70). Currently, the most used is Lutetium-177 (^177^Lu) labelling, which emits beta particles with approximately 1-mm path length to deliver radiation to sites of disease. A phase II trial investigating its use in metastatic castrate-resistant PC showed a PSA response rate of 96.7%, low toxic effects, and improvements in pain palliation (71). The most common toxic effects included xerostomia (87%), nausea (50%), and fatigue (50%), whilst a minority of patients (13%) experienced thrombocytopenia. Current active studies include the Australian LuPARP (NCT03874884) and American NCT03805594, which assess the efficacy of ^177^Lu in combination with targeted therapy or immunotherapy, whilst promising preliminary results are seen in trials such as TheraP (NCT03392428), which investigates its efficacy compared with conventional chemotherapy (72). Additionally, for castrate-sensitive PC, the international PSMAddition (NCT04720157) and Australian UpFrontPSMA (NCT04343885) (73) are large randomised trials evaluating the efficacy of ^177^Lu-PSMA-617 compared with the standard of care. The Australian POPSTAR II (NCT PENDING) phase II trial also aims to investigate the castrate-sensitive PC group. It aims to investigate patients with <5 metastases and compare SABR with or without Lu-PSMA.

Furthermore, trials (**Table 2**) for PSMA PET in OMPC patients include the use of radiopharmacy, SBRT, and SLND. A phase II trial from the Netherlands (NCT04443062) aims to compare ^177^Lu to delayed ADT in patients with BCR [^18^F-PSMA PET-CT-positive metastases in bones and/or lymph nodes (≤5 metastases)] and inability to perform local treatment for oligometastases (74). An American phase I trial (NCT05079698) combining SBRT and ^177^Lu infusion in PSMA PET-detected lesions (≤3 metastases) aims to identify the dose-limiting toxicity in their pilot study. The DETECT trial (NCT04300673) provides an exciting take on SLND in patients with ≥1 ^18^F/^68^Ga-PSMA-PET/CT suspected positive metastasis pelvic lymph nodes. All patients receive ^111^Indium (^111^In) PSMA tracer 24 h prior to surgery with the aim to evaluate the feasibility of ^111^In guided detection of lymph node metastases with intra-operative gamma-probe. Finally, a phase II trial (NCT03525288) comparing PSMA PET-guided definitive RT to standard care RT without PSMA PET aims to identify failure-free survival.

**Table 2:** Current active trials for PSMA PET-guided MDT in oligometastatic prostate cancer.

Name (trial number)	Location	Phase	Abbreviated Oncologic Eligibility	Treatment arms
Early Prostate Cancer Recurrence With PSMA PET Positive Unilateral Pelvic Lesion(s) (ProsTone) (NCT04271579)	Hamburg, Germany	–	-Hormone-sensitive PC recurrence after RP -Unilateral detection of ≤3 PSMA PET positive lymph node metastases in the pelvis -PSA at the time of PSMA PET < 4ng/ml	-Unilateral lymph node dissection (PSMA PET positive side) -Bilateral lymph node dissection
Multicentre Randomised Trial of ^68^Ga-PSMA-11PET/CT Based SRT after Radical Prostatectomy (PSMA SRT) (NCT03582774)	California, United States of America	III	-Planned SRT for recurrence after radical prostatectomy -PSA ≥0.1 ng/ml	-Standard of care SRT -^68^Ga-PSMA-11 PET/CT with subsequent SRT
Fluciclovine F18 or Ga68-PSMA PET/CT to Enhance Prostate Cancer Outcomes (NCT03762759)	Georgia, United States of America	II	-Post radical prostatectomy -Detectable PSA -No skeletal or systemic (extra-pelvic) metastases -Willingness to undergo pelvic RT	-Fluciclovine F18 PET/CT -^68^Ga-PSMA PET/CT
Lutetium-177-PSMA-617 in Oligometastatic Hormone Sensitive Prostate Cancer (NCT04443062)	Amsterdam, Netherlands	II	-Biochemical recurrence (PSA >1.0 ng/L) -PSA-doubling time <6 months -^18^F-PSMA-PET-CT-positive metastases in bones and/or lymph nodes (max. 5 metastases) -Unable to have local treatment for oligo-metastases -No prior hormonal therapy/taxane-based chemotherapy	-^177^Lu-PSMA radioligand therapy -Deferred ADT (however, control arm can receive ^177^Lu-PSMA in case of disease progression
Radio Guided Lymph Node Dissection in Oligometastatic Prostate Cancer Patients (DETECT) (NCT04300673)	Gelderlands, Netherlands	I/II	-≥1 ^18^F/68Ga-PSMA PET/CT suspected positive metastasis pelvic lymph nodes -Suitable for pelvic lymph node dissection	-All patients receive ^111^In-PSMA tracer 24 h prior to pelvic lymph node dissection
A Study of Stereotactic Body Radiotherapy (SBRT) and ^177^Lu-PSMA-617 for the Treatment of Prostate Cancer (NCT05079698)	New York, United States of America	I	-Previous treatment with surgery and/or definitive radiation ≥2 years prior -1–3 oligometastatic tumours detectable on PSMA PET -Lesions must be amenable to SBRT to a dose of 9 Gy 3× -PSA ≥0.5ng/ml but ≤50 ng/ml	-^177^Lu-PSMA-617 intravenous infusion with SBRT after the 2nd cycle
PSMA-PET Guided Radiotherapy (PSMA-PETgRT) (NCT03525288)	Quebec, Canada	II/III	-High risk of distant metastasis defined by any of: oligometastasis (≤5) (regional or distant) identified on conventional staging, newly diagnosed high-risk localized prostate cancer and CAPRA score 6–10, prior history of treated prostate cancer (RP or RT) and biochemical failure	-PSMA-PETgRT, PSMA PET during treatment planning, all lesions (≤5) treated with definitive RT -No PSMA PET/CT as part of RT treatment planning

The advent of PSMA PET has been paradigm shifting in the world of PC, which has propagated an exciting field of discovery. From its use as a diagnostic tool and identification of early BCR to PSMA-targeted therapies, the current landscape of MDT in OMPC is promising. However, PSMA PET in the setting of MDT is still in its infancy and is not ready for prime time. We therefore eagerly await the results of upcoming clinical trials.

## Author contributions

MA: draft preparation. AY: draft preparation. NP: draft revision. SS: draft revision. JI: draft revision, supervision. KT: draft revision, supervision. JE: draft revision, supervision. DB: draft revision, supervision, funding. MP: draft revision, supervision. All authors contributed to the article and approved the submitted version.

## Funding

This work was supported by the Sidney Kimmel Center for Prostate and Urologic Cancers at MSK and the NIH/NCI Cancer Center Support Grant to Memorial Sloan Kettering Cancer Center (P30 CA008748). Marlon Perera is sponsored by the Australian–America Fulbright Commission administered through a 2021–2022 Fulbright Future Scholarship funded by The Kinghorn Foundation.

## Conflict of interest

The authors declare that the research was conducted in the absence of any commercial or financial relationships that could be construed as a potential conflict of interest.

## Publisher’s note

All claims expressed in this article are solely those of the authors and do not necessarily represent those of their affiliated organizations, or those of the publisher, the editors and the reviewers. Any product that may be evaluated in this article, or claim that may be made by its manufacturer, is not guaranteed or endorsed by the publisher.
